# Diagnosis and therapy of myasthenia gravis—the patients’ perspective: a cross-sectional study

**DOI:** 10.3389/fneur.2023.1214041

**Published:** 2023-08-04

**Authors:** Tomasz Sobierajski, Anetta Lasek-Bal, Marek Krzystanek, Nils E. Gilhus

**Affiliations:** ^1^The Center of Sociomedical Research, Faculty of Applied Social Sciences and Resocialization, University of Warsaw, Warsaw, Poland; ^2^Department of Neurology, School of Health Sciences, Medical University of Silesia in Katowice, Katowice, Poland; ^3^Department of Neurology, Upper-Silesian Medical Center of the Medical University of Silesia in Katowice, Katowice, Poland; ^4^Department and Clinic of Psychiatric Rehabilitation, Faculty of Medical Sciences in Katowice, Medical University of Silesia in Katowice, Katowice, Poland; ^5^Department of Clinical Medicine, University of Bergen, Bergen, Norway; ^6^Department of Neurology, Haukeland University Hospital, Bergen, Norway

**Keywords:** corticosteroids, thymoma, physical health, mental health, Poland

## Abstract

The survey aimed to explore patients’ perspectives with myasthenia gravis (MG) toward the diagnosis made and the therapy used to treat MG. The survey was conducted with a quantitative method, using the CAWI technique. A total of 321 people participated in the survey. More than half of the respondents (56.4%) had suffered from MG for less than 10 years. In three out of 10 cases (30.9%), the diagnosis of MG lasted 3 years or longer. The diagnostic delay was significantly longer in female respondents than in the males (*p* = 0.029). Cholinergic drugs were used in 92.9% of cases initially, and as maintenance therapy in 84.3% of cases. Corticosteroids were used in initiating therapy (45.8%) and as maintenance therapy (46.4%). One in four respondents (25.5%) reported experiencing very strong and strong side effects after using steroids. The side effects from steroid therapy very strong or strong affected overall physical health in 55.9% of respondents, very strong or strong affected self-acceptance in 52%, to a very large or large extent on mental health in 47.1%, and to a very strong or strong extent influenced the performance of daily activities in 28.2%. More than half of the respondents (57.0%) had had a thymectomy. Seven out of 10 respondents (72.0%) declared that the therapy they were on at the time of the survey allowed them (to varying degrees) to control their course of MG. Low therapy acceptance and less well controlled MG was associated with a preference for non-tablet therapies (*p* = 0.045). Regular follow-up and cooperation with the specialist health care system should improve MG symptoms, activities of daily living, and quality of life.

## Introduction

Myasthenia gravis (MG) is an autoimmune disease with an annual incidence rate of 5–30 people per million (1–4). It is the most common neuromuscular transmission disorder, primarily affecting adults (5–7). Its prevalence has increased in recent decades as detection and survival rates have improved (8, 9). Although the exact cause of MG is unknown, the thymus appears to play a role (10, 11). Viral and bacterial infections have been suggested to initiate the immune response (12, 13). Additionally, thyroid disease is present in 3–8% of MG patients, and tumors are found in the thymus of 10–15% of patients (8, 14). A typical symptom is general muscle weakness affecting the bulbar muscles and those responsible for limb and eye movements (15–17).

Myasthenia gravis is diagnosed based on a detailed history, clinical examination, and laboratory testing (18). Sometimes, however, it can take a long time to diagnose the disease, and the availability of the tests conducted to detect MG can vary depending on regional and institutional conditions (19). MG therapy can take on various forms—more or less invasive from the patient’s perspective (20–22).

Understanding patients’ attitudes toward therapy and diagnosis allows medical personnel to respond appropriately through drug therapy and other therapeutic modalities, improving disease management and patients’ quality of life (23, 24). Factors affecting patients’ attitudes toward the disease include access to new medications, such as biologics, and access to non-medical sources of information.

Our study aimed to explore the perspectives of patients with MG concerning the diagnostic process and the treatment administered at the time of diagnosis, and to assess the impact of the therapy administered on patients’ well-being.

## Materials and methods

### Study design

The survey was a sociomedical study, combining interdisciplinary thinking on the interaction between social factors, demographics, and MG sufferers’ perspectives on diagnosis and therapy. The survey was conducted among people with MG in November/December 2022 in Poland.

### Population and sample size

The number suffering from MG in Poland is estimated to be about 8,000 people (25). Our study included 321 adults with MG, representing 0.05% of the total number in Poland. It was impossible to use a sampling frame for our survey, and the study is unrepresentative from the point of view of random sampling.

### The questionnaire

For this study, a survey instrument was created to find answers to the research questions and to achieve our research objective. The survey consisted of 38 questions. Four questions were metrics; they asked about gender, age, education, and place of residence. The remaining questions were pertinent to the research objective. The survey questions consisted of three thematic sections: diagnosis of MG, therapy of MG, and psychosocial aspects of MG. Most of the questions were closed, and a few were open-ended. Closed questions were dichotomous, questions with a scale, or questions with an option to choose one or more answers. Questions with scales were dominated by variants of the Likert scale, arranged on a verbal or numerical axis to explore attitudes toward a given phenomenon.

The survey instrument was prepared due to multi-stage consultations with doctors and people with MG. The tool was validated *ad hoc* by specialists in the field. A pilot study was conducted including eight people with MG to evaluate the tool methodologically. Data from the pilot study were not included in the actual study.

### Data collection procedure

The questionnaire was formally adapted to the requirements of the Computer Assisted Web Interviewing (CAWI) technique, with the help of which the survey was implemented. The questionnaire for the study was hung on Google’s cipher drive so that only the paper’s first author had access to all the data collected. At the same time, IP numbers or any other information identifying a specific person completing the questionnaire was not collected during the survey. The link with the survey questionnaire with instructions for self-completion was forwarded to the Polish Association of Myasthenia Gravis “Gioconda.” Representatives of the association sent the link to all members of their association. Eligibility for the survey required being an adult with MG and giving informed consent to participate in the study. The study included three individuals who completed the questionnaire on behalf of their children with MG; however, these responses were not included in the results pool for methodological and ethical reasons. Survey results were collected on disk.

### Statistical analysis

Statistical analyses were performed in IBM SPSS Statistics 28.0.1.0. Data for all outcomes are reported for all participants. The relationship between variables was evaluated by using the Chi-squared test. The Kruskal-Wallis’ test was used to analyze the questions using the Likert scale. Answers to questions are presented with a total number of respondents (*n*) and frequencies (%). For all analyzes, a *p* level of <0.05 was considered statistically significant.

### Ethical considerations

In implementing the survey, care was taken to ensure that it complied with the ethical rules of conducting social research. Only adults (age > 18 years) participated in the survey, and each gave informed consent to complete the survey. To ensure the respondents’ sense of confidentiality and anonymity in the subsequent analysis, all surveys were coded on the platform collecting the results. Only one person had access to the coded results, so it was impossible to link a person to specific results in the analysis process. The survey results are analyzed collectively, and identification at the data reporting level of a specific surveyed person is also impossible. The study received Silesia Medical University Ethics Committee’s approval (no. BNW/NWN/0052/KB/61/23).

## Results

Most of the respondents were women. More than a half of respondents (56.4%) had suffered from MG for less than 10 years (Table 1).

**Table 1:** Sociodemographic characteristics of respondents (*N* = 321).

	*N* (%)
Sex	
Female	288 (89.7)
Male	33 (10.3)
Age	
18–30	39 (12.1)
31–40	87 (27.1)
41–50	100 (31.2)
51–60	65 (20.2)
61–70	21 (6.5)
71 and more	9 (2.8)
Education	
Primary	8 (2.5)
Vocational	34 (10.6)
Secondary	116 (36.1)
Tertiary	163 (50.8)
Place of residence	
Village < 1,000 residents	56 (17.4)
Village > 1,000 residents	38 (11.8)
City < 10 K residents	25 (7.8)
City 10–50 K residents	65 (20.2)
City 51–200 K residents	56 (17.4)
City 201–500 K residents	29 (9.0)
City 501 K—1 million residents	33 (10.3)
City > 1 million residents	19 (5.9)
Length of MG from diagnosis	
< 1 year	34 (10.6)
2–5 years	85 (26.5)
6–10 years	62 (19.3)
11–15 years	45 (14.0)
16–25 years	56 (17.4)
>26 years	39 (12.1)

### Myasthenia gravis diagnosis

Half of respondents (47%) had been diagnosed with MG before age 30 years, often after a long delay (Table 2).

**Table 2:** Myasthenia gravis diagnosis process (*N* = 321).

Age of diagnosis of MG	
Childhood	21 (6.5)
18–30 years old	130 (40.5)
31–40 years old	75 (23.4)
41–50 years old	58 (18.1)
51–60 years old	24 (7.5)
61–70 years old	10 (3.1)
71 and more years old	3 (0.9)
Number of years between first symptoms and diagnosis MA	
<1 year	142 (44.2)
1–2 years	80 (24.9)
3–10 years	86 (26.8)
>10 years	13 (4.1)
Circumstances of diagnosis of MG	
By chance, because of routine examinations	20 (6.2)
After miastenic crisis	23 (7.2)
The doctor immediately suspected MA and conducted tests for it	94 (29.3)
After a long search for the cause	184 (57.3)
Miasthenic crisis	
Yes	143 (44.5)
No	178 (55.5)
Self-evaluation of the MG’s diagnosis process	
The diagnosis process was very short and not very complicated	93 (29.0)
The diagnosis process was short but very complicated	60 (18.7)
The diagnosis process was long, though not very complicated	60 (18.7)
The diagnosis process was very long and very complicated	108 (33.6)

Our MG group included more females in the younger group and more males in the elderly group (*p* < 0.001). MG was diagnosed more often due to myasthenic crisis in the youngest age group (*p* = 0.002; Supplementary Table S1).

The diagnostic delay was significantly longer in female respondents than in the males (*p* = 0.029). The physician’s suspicion of MG right away and conducting specific tests for it was significantly more common in males than in females (*p* = 0.029; Table 3).

**Table 3:** Circumstance of diagnosis of MG vs. sociodemographic characteristics of respondents (*N* = 321).

	Circumstances of diagnosis				*p* value
	By chance, because of routine examinations	After miastenic crisis	The doctor immediately suspected MA and conducted tests for it	After a long search for the cause	
	*N* (%)				
Sex					
Female	15 (5.2)	20 (6.9)	81 (28.1)	172 (59.7)	0.029
Male	5 (15.2)	3 (9.1)	13 (39.4)	12 (36.4)	
Age					
18–30	2 (5.1)	2 (5.1)	12 (30.8)	23 (59.0)	< 0.001
31–40	3 (3.4)	6 (6.9)	20 (23.0)	58 (66.7)	
41–50	5 (5.0)	9 (9.0)	31 (31.0)	55 (55.0)	
51–60	5 (7.7)	0 (0.0)	25 (38.5)	35 (53.8)	
61–70	2 (9.5)	5 (23.8)	2 (9.5)	12 (57.1)	
71 and more	3 (33.3)	1 (11.1)	4 (44.4)	1 (11.1)	
Education					
Primary	1 (12.5)	1 (12.5)	0 (0.0)	6 (75.0)	0.373
Vocational	3 (8.8)	1 (2.9)	11 (32.4)	19 (55.9)	
Secondary	10 (8.6)	10 (8.6)	37 (31.9)	59 (50.9)	
Tertiary	6 (3.7)	11 (6.7)	46 (28.2)	100 (61.3)	
Length of MG from diagnosis					
< 1 year	2 (5.9)	5 (14.7)	10 (29.4)	17 (50.0)	0.193
2–5 years	7 (8.2)	5 (5.9)	29 (34.1)	44 (51.8)	
6–10 years	6 (9.7)	5 (8.1)	19 (30.6)	32 (51.6)	
11–15 years	2 (4.4)	2 (4.4)	8 (17.8)	33 (73.3)	
16–25 years	2 (3.6)	4 (7.1)	11 (19.6)	39 (69.6)	
>26 years					
Age of diagnosis of MG					
Childhood	1 (4.8)	1 (4.8)	6 (28.6)	13 (61.9)	0.002
18–30 years old	3 (2.3)	11 (8.5)	36 (27.7)	80 (61.5)	
31–40 years old	4 (5.3)	2 (2.7)	26 (34.7)	43 (57.3)	
41–50 years old	4 (6.9)	3 (5.2)	16 (27.6)	35 (60.3)	
51–60 years old	4 (16.7)	2 (8.3)	8 (33.3)	10 (41.7)	
61–70 years old	3 (30.0)	3 (30.0)	1 (10.0)	3 (30.0)	
71 and more years old	1 (33.3)	1 (33.3)	1 (33.3)	0 (0.0)	

### Myasthenia gravis therapy

**Figure 1:**
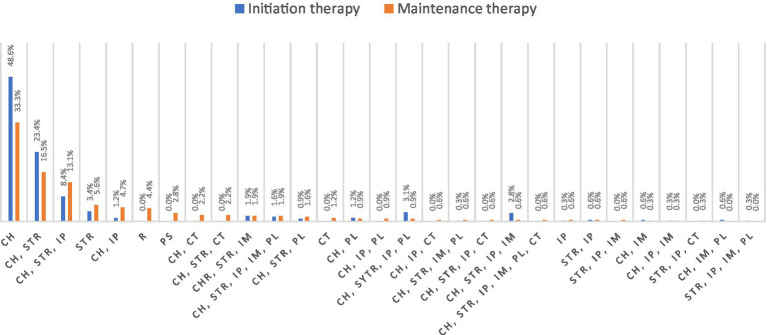
Distribution of initiating therapy and maintenance therapy in all included patients (*N* = 321). CH, cholinergic; STR, steroid therapy; IP, immunosuppressants; IM, immunoglobulins; PL, plasmapheresis; CT, clinical trial; PS, parasympathomimetics; and R, remission.

Corticosteroids and cholinergic drugs were the most frequently used drugs in initiating and maintaining therapy. The same percentage of patients stated that corticosteroids were used in initiating therapy (45.8%) and as maintenance therapy (46.4%). Cholinergic drugs were used in 92.9% of cases initially, and as maintenance therapy in 84.3% of cases. An overview of all therapeutic combination and monotherapies is shown in Figure 1.

One in four respondents (25.5%) reported experiencing very strong or strong side effects after using steroids. In 4.4% of cases, side effects were severe but passed quickly. In three out of 10 cases (30.2%), side symptoms after using steroids were minor but constant, and in 7.8%, they were minor and passed quickly. One in seven (13.1%) said they did not experience any side effects from steroid use. One in five patients (19%) had never taken steroids for MG treatment.

The side effects from steroid therapy very strong or strong affected overall physical health in 55.9% of respondents, very strong or strong affected self-acceptance in 52%, to a very large or large extent on mental health in 47.1%, and to a very strong or strong extent influenced the performance of daily activities in 28.2% (Table 4).

**Table 4:** Summary of answers to the questions: did steroid side effects cause a change in treatment? vs. did the use of steroid therapy affect specific areas of life? (*N* = 321).

Change of treatment	Degree of steroid therapy’s affect specific areas of life								
	Not at all	Very slightly	Slightly	Rather slightly	Rather strongly	Strongly	Very strongly	*p* value	SD
	*N* (%)								
Performing daily activities									
I did	14 (13.7)	12 (11.8)	7 (6.9)	14 (13.7)	16 (15.7)	14 (13.7)	25 (14.5)	< 0.001	2.299
I did not	52 (29.7)	26 (14.9)	20 (11.4)	15 (8.6)	19 (10.9)	15 (8.6)	28 (16.0)		
n/a	44 (100.0)	-	-	-	-	-	-		
Sens of self-acceptance									
I did	16 (15.7)	8 (7.8)	6 (5.9)	9 (8.8)	10 (9.8)	11 (10.8)	42 (41.2)	< 0.001	2.421
I did not	47 (26.9)	25 (14.3)	26 (14.9)	11 (6.3)	18 (10.3)	18 (10.3)	30 (17.1)		
n/a	44 (100.0)	-	-	-	-	-	-		
Mental health									
I did	13 (12.7)	10 (9.8)	10 (9.8)	5 (4.9)	16 (15.7)	15 (14.7)	33 (32.4)	< 0.001	2.332
I did not	49 (28.0)	20 (11.4)	27 (15.4)	18 (10.3)	17 (9.7)	19 (10.9)	25 (14.3)		
n/a	44 (100.0)	-	-	-	-	-	-		
General physical health beyond MG									
I did	11 (10.8)	8 (7.8)	5 (4.9)	10 (9.8)	11 (10.8)	25 (24.5)	32 (31.4)	< 0.001	2.313
I did not	44 (25.1)	24 (13.7)	22 (12.6)	27 (15.4)	15 (8.6)	20 (11.4)	23 (13.1)		
n/a	44 (100.0)	-	-	-	-	-	-		

One in eight respondents (12.5%) had experienced immunoglobulin therapy once or twice, and one in nine (11.5%) three or more times. Three-quarters of the patients (76.0%) had not had immunoglobulin therapy.

Plasmapheresis once or twice had been given to 6.9% of respondents, and 12.8% had plasmapheresis three or more times. Eight out of 10 respondents (80.4%) said they had never received plasmapheresis.

More than half of the respondents (57.0%) had had a thymectomy.

**Figure 2:**
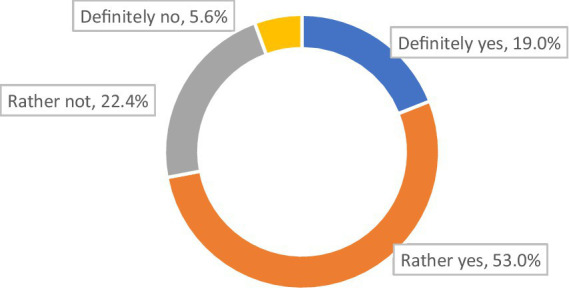
Does the therapy you are currently using control allow to control MG? (*N* = 321).

Seven out of 10 respondents (72.0%) declared that the therapy they were on at the time of the survey allowed them (to varying degrees) to control their course of MG (Figure 2).

Among those with no plasmapheresis, three-quarters (77.2%) stated that their present therapy [definitely (19.4%) or in part (57.8%)] allowed them to control the symptoms of their disease (*p* < 0.001). Among those who had plasmapheresis once or twice, four in 10 (40.9%) stated that their present therapy [definitely (22.7%) or in part (18.2%)] allowed them to control the disease (*p* < 0.001). For the group who had plasmapheresis three times or more, more than half (56.1%) stated that their therapy [definitely (14.6%) or in part (41.5%)] allowed them to control (to varying degrees) their disease (*p* < 0.001).

**Figure 3:**
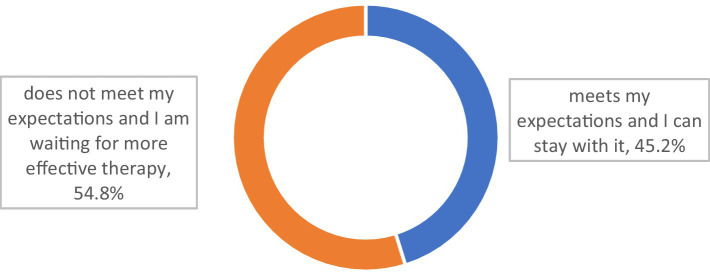
Does the therapy you are currently using meet your expectations? (*N* = 321).

More than half of the respondents (54.8%) declared that their present MG treatment did not meet their expectations, and they expected it to be modified or escalated (Figure 3).

Those who had immunoglobulin therapy were more likely to declare that their present therapy did not meet their expectations (*p* = 0.007). The trend was the same for those who had received plasmapheresis (*p* = 0.051; Table 5).

**Table 5:** Summary of responses to the question: does the current MG treatment therapy meet your expectations? vs. immunoglobulin treatment and interventional treatment (*N* = 321).

	Does the therapy you are currently using meet your expectations?		
	Therapy meets my expectations, and I can stay with it	Therapy does not meet my expectations and I am waiting for more effective therapy	*p* value
	*N* (%)		
Have you had immunoglobulin therapy?			
Neither once	122 (50.0)	122 (50.0)	0.007
Once or twice	13 (32.5)	27 (67.5)	
Three or more times	10 (27.0)	27 (73.0)	
Have you had plasmapheresis?			
Neither once	124 (48.1)	134 (51.9)	0.051
Once or twice	5 (22.7)	17 (77.3)	
Three or more times	16 (39.0)	25 (61.0)	
Have you had thymus removal surgery?			
I have	87 (47.5)	96 (52.5)	0.326
I have not	58 (42.0)	80 (58.0)	

**Figure 4:**
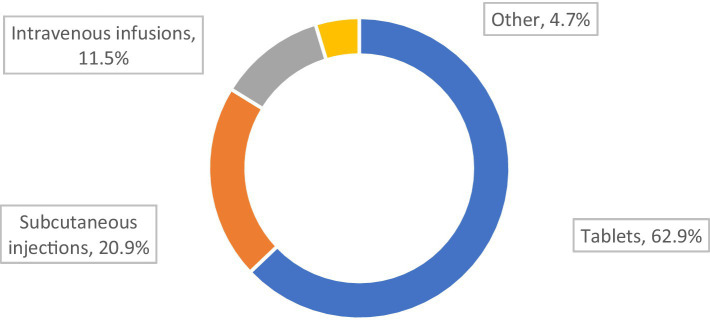
Preferred application form of the drugs (*N* = 321).

The most desirable form of drug administration for our MG respondents was tablets, and the least desirable was intravenous infusions (Figure 4).

Low therapy acceptance and less well controlled MG was associated with a preference for non-tablet therapies (*p* = 0.045; Table 6).

**Table 6:** Summary of responses to the preferred application form of the drugs vs. question: does the therapy you are currently using allow to control MG? (*N* = 321).

	Preferred application form of the drugs				*p* value
	Subcutaneous injections	Pills	Intravenous infusions	Other	
	*N* (%)				
Does the therapy you are currently using allow to control MG?					
Definitely yes	11 (18.0)	45 (73.8)	3 (4.9)	2 (3.3)	0.045
Rather yes	31 (18.2)	110 (64.7)	21 (12.4)	8 (4.7)	
Rather no	19 (26.4)	39 (54.2)	11 (15.3)	3 (4.1)	
Definitely no	6 (33.3)	8 (44.4)	2 (11.1)	2 (11.1)	

A trend indicated that tablets represent the preferred form of drug administration by those who declared that their therapy met their expectations (*p* = 0.053) The patients who stated that the therapy did not meet their expectations were twice as likely to prefer subcutaneous for drug administration (*p* = 0.053).

Eight in 10 patients (81.2%) reported an additional burden of non-MG diseases; 16.8% reported one comorbidity, whereas 61.7% had two or more comorbidities. The most common comorbidities were depression, diabetes, hypertension, Hashimoto’s thyroiditis, and hypothyroidism.

## Discussion

In clinical practice, a neurologist periodically assesses the condition of MG patients by evaluating their clinical and functional status. Available scales make it possible to estimate the patient’s neurological status and severity of symptoms but usually do not consider the impact of the disease on the patients’ sociopsychological condition (23).

In the present study, patients completed questionnaires without the presence of their neurologist. To achieve optimal collaboration with the patient, it is necessary to determine their level of knowledge about their disease, its therapy, and their quality of life.

More than 60% of patients stated that the process preceding the diagnosis of MG was long and complicated. This may be surprising given that, in most cases, the first symptoms of MG are quite distinctive and include typical eye movement disturbances. However, such a diagnostic delay has been reported also in previous studies and reflects that MG is a rare disease. Most general practitioners have not seen the disease preciously. Within 3 years, about 90% of patients develop symptoms of generalized muscle weakness and fatigue (26), which can affect the daily acts of swallowing, chewing, breathing, and speaking (27). Interestingly, most of our study patients resided in large cities with relatively easy access to medical care specialists, but still they experienced diagnostic delay.

The chronic character of the disease and the variation of MG symptoms over time with a risk of severe exacerbations worsen the patients’ quality of life. Corticosteroid therapy may cause mental side-effects (28). Interestingly, in the present study, over 40% of patients believed they had experienced a myasthenic crisis. Many patients have probably mistaken it for a severe disease exacerbation, as epidemiological data do not indicate that myasthenic crisis is so common among MG patients (29). Crisis with a transient need of intensive care and respiratory support usually occurs in 15–20% of all MG patients and can be precipitated by infection, a stressful situation, a surgical procedure, or as a reaction to medication (8, 30). Modifiable risk factors for disease exacerbation should be identified, discussed with the patient, and treated.

According to the results of the present study, one fourth of the patients poorly tolerate steroids, and only 13% are free from adverse effects. Physical effects were reported by over half of the study population, and mental effects were reported by nearly one half. Many expressed having problems with self-acceptance.

Most patients did not undergo human immunoglobulin or plasmapheresis treatment, but every 10 underwent at least three courses of such therapies. The beneficial effects of such therapies last only for 3–4 months, and this may be the main reason why so many of our study patients found that this form of therapy failed their expectations. It is worth noting that immunosuppressants and their side effects can affect patients’ perspective (31) However, over half of those treated with plasmapheresis reported good disease management. The present study shows that most patients prefer taking tablets, despite potential swallowing problems as part of the disease. 20% of the study patients preferred subcutaneous drug administration, which may be relevant when planning future pharmacological policies.

Over half of the study patients had their thymus resected. Anxiety about a potential thymus tumor can affect patients’ well-being, as the incidence of thymoma is 10–15% of all MG patients (8, 32, 33). Surgical removal of the tumor and the good prognosis may reduce the prevalence of anxiety and depression among MG patients with thymoma (34, 35). The median 5-year survival rate is 69% for patients with advanced thymoma or thymic carcinoma (36).

The results presented in this article illustrate the need for further patient education. Patients’ adequate interpretation of disease symptoms improves their understanding and reduces their associated anxiety or stress. If a patient’s knowledge is inaccurate, they may want to intensify unnecessary therapies and thus become exposed to the adverse effects of drugs. They may also underreport MG-associated symptoms and thus miss useful therapy.

A strength of our study is that most of the patients have had MG for over 10 years. It illustrates long-term management and disease knowledge acquired by experience. Knowing patients’ views and attitudes toward the disease aids in therapy management and is an integral part of health technology assessment.

### Limitations of the study

The patients in our study were unequally distributed in terms of education, with a large proportion having higher education levels. Although our patients’ sample was large, simple random sampling was not used. Patients surveyed made self-assessments in many aspects of the study, which many factors could have influenced at the time of completion. Underrepresented in the survey are the oldest people, over 60. It was because the survey was conducted over the Internet, which for many older people is a significant barrier or a result of digital exclusion. Nevertheless, the results allow us to capture the main cognitive categories in diagnosing and treating MG patients in Poland.

## Conclusion and recommendations

Diagnostic delays and misconceptions about the myasthenic crisis were observed, indicating the necessity for improved patient education. A significant proportion of patients reported adverse effects of therapies, particularly corticosteroids, emphasizing the importance of personalized treatment approaches.

Considering the above research results, we recommend that healthcare professionals focus on enhancing patient education, helping patients better understand MG symptoms, interpret them accurately, and manage the condition effectively. It could reduce unnecessary therapies and improve treatment outcomes. It is essential to raise awareness among general practitioners about the distinct symptoms of MG, aiming to minimize diagnostic delays. Early referral to specialists can ensure prompt diagnosis and timely initiation of treatment. Treatment approaches should be optimized by considering patient preferences. Factors such as the mode of drug administration (e.g., tablets or subcutaneous delivery) should be considered. Personalized approaches can minimize adverse effects and enhance treatment adherence. We think there is a need to educate patients about myasthenic crises, providing accurate information about their nature and prevalence. It will help prevent unnecessary anxiety and inappropriate treatment-seeking behaviors.

Implementing these recommendations will support a patient-centered approach to managing MG, considering patients’ overall well-being. It will improve treatment outcomes and an enhanced quality of life for individuals with MG.

## Data availability statement

The raw data supporting the conclusions of this article will be made available by the authors, without undue reservation.

## Ethics statement

The studies involving human participants were reviewed and approved by Bioethic Commitee of Medical University of Silesia. The patients/participants provided their informed consent to participate in this study.

## Author contributions

TS designed the study, conceptualized the methodology, and performed data curation, formal analysis, and statistical analysis. TS, AL-B, and NG wrote original draft and edited and revised the manuscript. NG and MK supervised the manuscript. All authors contributed to the article and approved the submitted version.

## Conflict of interest

The authors declare that the research was conducted in the absence of any commercial or financial relationships that could be construed as a potential conflict of interest.

## Publisher’s note

All claims expressed in this article are solely those of the authors and do not necessarily represent those of their affiliated organizations, or those of the publisher, the editors and the reviewers. Any product that may be evaluated in this article, or claim that may be made by its manufacturer, is not guaranteed or endorsed by the publisher.
