# Supplementary material for: Diagnosis and therapy of myasthenia gravis—the patients’ perspective: a cross-sectional study
Source: Front Neurol. 2023 Aug 4;14:1214041. doi: 10.3389/fneur.2023.1214041 (PMC10437051; doi:10.3389/fneur.2023.1214041)
Supplement: Supplementary file 1 [file Table_1.docx]

**Table 1S**. Age of diagnosis of MG vs. Sociodemographic characteristics of respondents (N = 321)

|  | Age diagnosis of MG | | | | | | |  |
| --- | --- | --- | --- | --- | --- | --- | --- | --- |
|  | 18-30 | 31-40 | 41-50 | 51-60 | 61-70 | 71-80 | Childhood | *P* - value |
|  | N(%) | | | | | | |  |
| Sex | | | | | | | | |
| Female | 121(42.0) | 70(24.3) | 54(18.8) | 16(5.6) | 5(1.7) | 2(0.7) | 20(6.9) | < 0.001 |
| Male | 9(27.3) | 5(15.2) | 4(12.1) | 8(24.2) | 5(15.2) | 1(3.0) | 1(3.0) |  |
| Age | | | | | | | | |
| 18-30 | 36(92.3) | - | - | - | - | - | 3(7.7) | < 0.001 |
| 31-40 | 47(54.0) | 31(35.6) | - | - | - | - | 9(10.3) |  |
| 41-50 | 36(36.0) | 26(26.0) | 34(34.0) | - | - | - | 4(4.0) |  |
| 51-60 | 8(12.3) | 12(18.5) | 23(35.4) | 17(26.2) | - | - | 5(7.7) |  |
| 61-70 | 3(14.3) | 4(19.0) | 0(0.0) | 7(33.3) | 7(33.3) | - | 0(0.0) |  |
| 71 and more | 0(0.0) | 2(22.2) | 1(11.1) | 0(0.0) | 3(33.3) | 3(33.3) | 0(0.0) |  |
| Education | | | | | | | | |
| Primary | 3(37.5) | 2(25.0) | 0(0.0) | 0(0.0) | 1(12.5) | 1(12.5) | 1(12.5) | 0.009 |
| Vocational | 13(38.2) | 12(35.3) | 6(17.6) | 1(2.9) | 2(5.9) | 0(0.0) | 0(0.0) |  |
| Secondary | 45(38.8) | 21(18.1) | 23(19.8) | 14(12.1) | 6(5.2) | 0(0.0) | 7(6.0) |  |
| Tertiary | 69(42.3) | 40(24.5) | 29(17.8) | 9(5.5) | 1(0.6) | 2(1.2) | 13(8.0) |  |
| Length of MG from diagnosis | | | | | | | | |
| < 1 year | 7(20.6) | 12(35.3) | 9(26.5) | 3(8.8) | 2(5.9) | 1(2.9) | 0(0.0) | < 0.001 |
| 2-5 years | 28(32.9) | 19(22.4) | 22(25.9) | 9(10.6) | 5(5.9) | 2(2.4) | 0(0.0) |  |
| 6-10 years | 18(29.0) | 17(27.4) | 18(29.0) | 7(11.3) | 2(3.2) | 0(0.0) | 0(0.0) |  |
| 11-15 years | 25(55.6) | 10(22.2) | 6(13.3) | 2(4.4) | 0(0.0) | 0(0.0) | 2(4.4) |  |
| 16-25 years | 34(60.7) | 12(21.4) | 0(0.0) | 1(1.8) | 1(1.8) | 0(0.0) | 8(14.3) |  |
| >26 years | 18(46.2) | 5(12.8) | 3(7.7) | 2(5.1) | 0(0.0) | 0(0.0) | 11(28.2) |  |
| Circumstances of diagnosis of MG | | | | | | | | |
| By chance, because of routine examinations | 3(15.0) | 4(20.0) | 4(20.0) | 4(20.0) | 3(15.0) | 1(5.0) | 1(5.0) | 0.002 |
| After MG breakdown | 11(47.8) | 2(8.7) | 3(13.0) | 2(8.7) | 3(13.0) | 1(4.3) | 1(4.3) |  |
| The doctor immediately suspected MA and conducted tests for it | 36(38.3) | 26(27.7) | 16(17.0) | 8(8.5) | 1(1.1) | 1(1.1) | 6(6.4) |  |
| After a long search for the cause | 80(43.5) | 43(23.4) | 35(19.0) | 10(5.4) | 3(1.6) | 0(0.0) | 13(7.1) |  |
